# Endosulfan Elimination Using Amine-Modified Magnetic Diatomite as an Adsorbent

**DOI:** 10.3389/fchem.2022.907302

**Published:** 2022-05-26

**Authors:** İhsan Alacabey

**Affiliations:** Vocational Higher School of Healthcare Studies, Mardin Artuklu University, Mardin, Turkey

**Keywords:** adsorption isotherms, endosulfan, magnetic diatomite, pesticide, silane

## Abstract

Pesticides are among the most dangerous developing toxins since they are very hazardous to the environment and threaten human health. In this study, researchers successfully manufactured surface-modified magnetic diatomite (m-DE-APTES) and used them as a sorbent to extract endosulfan from an aqueous solution. There is no other study like it in the scholarly literature, and the results are astounding. Fourier transform infrared spectroscopy (FTIR), scanning electron microscopy (SEM), energy dispersive X-ray (EDX), electron spin resonance (ESR), and surface area measurements were used to analyze magnetic diatomite particles with surface modification. According to the analysis results, magnetic diatomite has a wide surface area and a porous structure. Furthermore, m-DE-APTES has a higher endosulfan adsorption capacity (97.2 mg g^−1^) than raw diatomite (DE) (16.6 mg g^−1^). Adsorption statistics agree with Langmuir adsorption isotherm (*R* ^2^ = 0.9905), and the adsorption occurred spontaneously at −2.576 kj mol^−1^ in terms of ΔG^o^. Finally, m-DE-APTES are a viable alternative adsorbent for removing pesticides from aqueous solutions.

## Introduction

Pesticides are the most frequently encountered persistent organic pollutants (POPs) in the environment (Ali and Jain, 1998; Basheer and Ali, 2018; Matsushita et al., 2018; Al-Shaalan et al., 2019; Xie et al., 2020). Pesticide residues can stay in the ecosystem for an extended period, contaminating the food chain (Marican and Durán-Lara, 2018; Kupski et al., 2019). The use of fungicides and pesticides is hazardous to health (Karanasios et al., 2012). The physiological reactions can cause harm to both target and non-target organisms and severe adverse effects on humans (Hernández et al., 2013). Certain pesticides are classified as potentially carcinogenic and cytotoxic agents because they have been linked to neurological disorders, infertility, bone marrow, immunological and respiratory diseases (Chawla et al., 2018). Even at low concentrations (ppm, ppb), these contaminants in the environment attract researchers’ attention.

According to data released by United Nations Environment Program and World Health Organization (WHO), Millions of people involved in agriculture in developing countries are exposed to the severe toxicity of the pesticide; as a result, approximate 18.000 people die each year (Ghuffar et al., 2021).

Pesticide removal techniques such as ozonation, hydrostatic pressure, and adsorption approaches have been widely employed (Chen et al., 2013a; Iizuka et al., 2013; Rasolonjatovo et al., 2017). Additionally, various adsorbents such as cryogels, monoliths, particles (magnetic, non-magnetic) and membranes (for filtration) have been used as an effective alternative adsorbents for the removal of organic pollutants from aqueous medium (Çorman et al., 2017; Acet et al., 2018; Armutcu et al., 2019). Furthermore, agricultural wastes such as silica and clay minerals (e.g., montmorillonite, zeolite, bentonite and kaolinite) have been employed as a cost-effective adsorbent to remove pesticides (Gimsing et al., 2007; Donia et al., 2012; Arnnok and Burakham, 2014).

Diatomite (DE) is one of the adsorbents of low-density mineral clays mainly consisting of hydrated silicon dioxide (SiO2.nH2O). This structure has garnered significant attention due to its large internal surface area, porosity, easy accessibility and eco-compatibility (Wu et al., 2013). In addition, its higher cation-exchange capacity (50 meq/100 g), larger surface area, porous structure, and chemical inertness make diatomites promising adsorbents (Tsai et al., 2006). DE is also used to filtrate microbial contamination such as bacteria, viruses, and protozoa and remove heavy metals from food, beverage, drinking water, surface and groundwaters (Robertson et al., 2003; Kul et al., 2010; Yuan et al., 2010; Michen et al., 2012; Kabiri et al., 2015; Johnson et al., 2020).

Applications of magnetic separation techniques using magnetic particles and their improvement have sparked researchers’ interest in recent years (Köse et al., 2015; Erol et al., 2016). Using natural materials with magnetic properties provides several advantages in terms of specific affinity, better selectivity, more simplicity, and higher adsorption rates. Each step of the separation process can be performed in a sample container or a test tube without the use of any sophisticated chromatographic techniques or expensive equipment. It is also possible to efficiently extract suspended solids directly from crude samples (Zheng et al., 2017). Their magnetic properties allow them to easily separate/decant from the adsorption medium by introducing an external magnetic field, i.e. permanent magnet (Edathil et al., 2018). Additionally, the magnetic separations’ capability and efficiency make them particularly well-suited for large-scale systems and adaptable to continuous separation systems (Köse et al., 2015).

Magnetic diatomite (m-DE) was synthesised, and afterwards, the modification was done with (3-aminopropyl) triethoxysilane (APTES) in this study. Later, this structure was used in pesticide adsorption, with endosulfan being the preferred pesticide. Since endosulfan contains electro-negative groups that can easily interact with the amino groups in the APTES molecule, it was particularly desirable to work with this pesticide. Concurrently, endosulfan is one of the most dangerous endocrine disruptors (Reynoso Varela et al., 2021). A similar study on modifying the synthesised m-DE with APTES and using this material to remove pesticides from aqueous systems has not been found in the literature. This aspect of the study also demonstrates how diatomite modified with silane structures can be used in various fields (Mu et al., 2018; Kucuk et al., 2020). However, when the advantages of magnetic separation were considered, a study with exciting results emerged.

## Materials and Methods

### Materials

Endosulfan (3-aminopropyl)triethoxysilane (APTES), ferric chloride hexahydrate (FeCl_3_.6H_2_O), ferrous chloride tetrahydrate (FeCl_2_.4H_2_O), hydrochloric acid (HCl), ethanol (absolute, 99%), sodium chloride (NaCl), and ammonium hydroxide solution (concentrated, 25%, w/V) were purchased from Sigma Aldrich (St. Louis, United States).

DE samples were gathered from the Çaldıran district of Van province. DE was extracted about 5 cm deep from the surface to prevent contaminations originating from the surface. The DE was granulated and sieved at 230 mesh. After mixing 100 g of DE inside pure water (1.7 L) on the rotator at 200 rpm for 10 h, it was allowed to form sediment for 12 h to separate the solid phase from the aqueous phase. The supernatant was removed, and precipitant (DE) was stored at 25°C until completely dry.

### Synthesis of Amine-Modified Magnetic Diatomite (m-DE-APTES)

**FIGURE 1:**
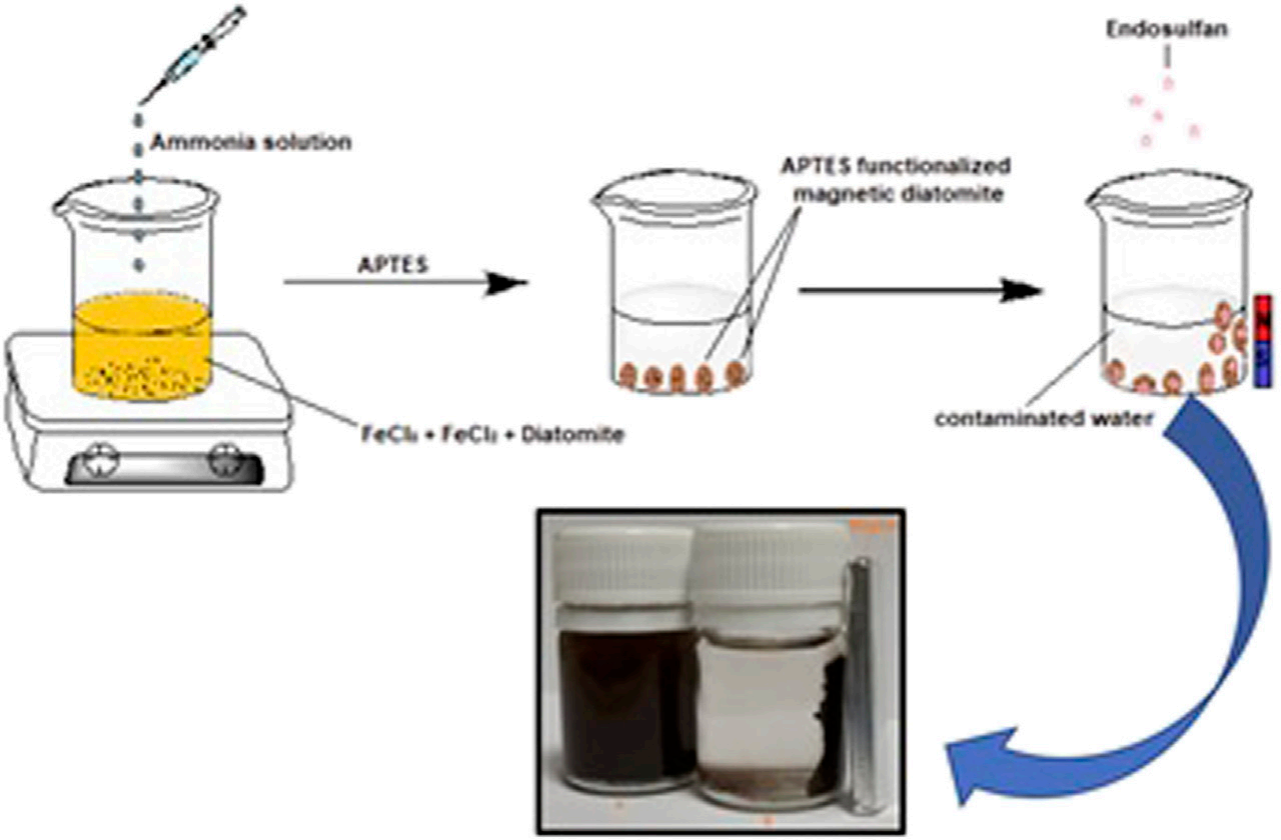
Schematic presentation of the synthesis of m-DE-APTES.

Before modifying DE with APTES, magnetic-DE was synthesised using the co-precipitation method as previously described (Massart, 1981). Herein, 4.093 g of FeCl_3_.6H_2_O and 2.595 g of FeCl_2_.4H_2_O salts were separately dissolved in 40 ml distilled water at 70°C, followed by the addition of 3 g diatomite (DE) to this solution. Afterwards, 0.7 M NaOH solution (500 ml) was dropped to precipitate DE coated magnetic particles at 25°C for 4 h. The magnetic-DE produced was subjected to a magnet and washed several times with distilled water to remove non-magnetic particles and unreactive salts.

m-DE (1.5 g) was dispersed in APTES solution (5 ml, 2%, w/V) produced using absolute ethanol, followed by vigorous stirring for 2 h to introduce charged functionalities. The resulting m-DE-APTES were washed with distilled water/absolute ethanol three times and then left to dry at 25°C and stored in a desiccator until use. Figure 1 shows a schematic presentation of the synthesis of m-DE-APTES employed in the study.

### Characterizations

To analyse the chemical structure of the particles, Fourier transform infrared spectroscopy (FTIR; instrument Thermo Nicolet iS10 FTIR Spectrometer, United States) was utilized in a wavenumber range between 4,000–500 cm^−1^. Scanning electron microscopy (SEM; Carl Zeiss AG-EVO^®^ 50 Series, Germany) was used to obtain particles’ morphologies, while Energy Dispersive X-Ray (SEM/EDX) analysis determined the elemental composition of structure semi-quantitatively. Electron spin resonance (ESR; Bruker ELEXSYS E580, China) device was used to determine the particles’ magnetic properties. Material is subjected to a magnetic field, which induces orientations in the electron spin. The magnitude of the magnetic field and the material’s temperature result in diverse spin orientations. The g factor, the spectroscopic cleavage, is calculated according to the following equation (Araujo et al., 2015).

$$g=\;\frac{h\upsilon }{\beta H_{r}}$$ (1)

Where h is the Planck constant (6.626 × 10^−27^ erg s); *β* is the Bohr magneton (×9.274 10^−21^ erg G^−1^); *ν* is the frequency (9.707 × 10^9^ Hz), and Hr is the resonance of magnetic field (G).

Specific surface area analysis was measured using a surface analyzer (Micromeritics TriStar II, United States) by applying the Brunauer–Emmett–Teller (BET) model (using N_2_ adsorption) (Mohan et al., 2020).

### Batch Adsorption Studies

The magnetic particles were used for evaluating their endosulfan adsorption performances. Endosulfan solution (5 ml) was placed in a test tube for the adsorption assay, and a 200 μl aliquot was isolated to determine the initial amount of endosulfan. A rotator was used to initiate adsorption after transferring 20 mg of the particle (m-DE-APTES, DE-APTES) to the test tube. The concentration of the endosulfan in the supernatant was measured at a wavelength of 212 by Spectrophotometry (TU-1810 UV-VIS Spectrophotometer, Pgeneral, China). The adsorption capacity was estimated according to the given equation (Erol, 2016; Erol et al., 2019a).

$$q_{e}=\left[\left(C_{0}-C_{e}\right)\;x\;V\right]/w$$ (2)

Here, *q*_*e*_: the equilibrium adsorption capacity (mg endosulfan/g particle), *C*_*o*_ and *C*_*e*_: the initial and final concentration of the endosulfan solution respectively (mg L^−1^), V: volume of adsorption medium (L) and w: the mass of the dry particle (g). 1 mol L^−1^ NaCl 10 ml solution was used for desorption agents. The particles adsorbed endosulfan were mixed with a rotator for 1 h in NaCl solution to ensure complete desorption. The following formula was used to calculate the desorption efficiency:

$$\text{Desorption}\;\text{efficiency}\;=\frac{\text{amount}\;\mathrm{of}\;\text{endosulfan}\;\text{desorbed}}{\text{amount}\;\mathrm{of}\;\text{endosulfan}\;\text{adsorbed}}\text{x}\;100$$ (3)

## Results and Discussions

### Characterization of the Adsorbents

**FIGURE 2:**
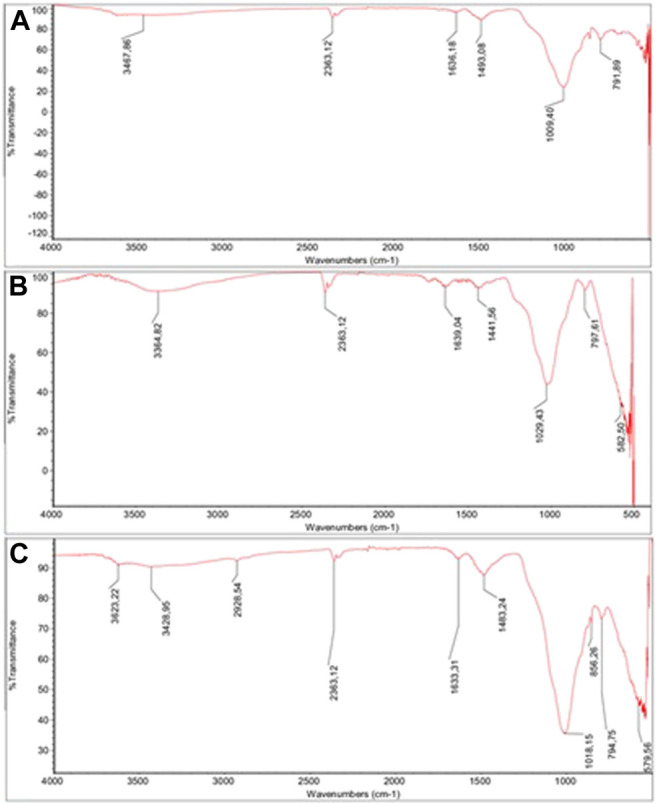
**(A)** FTIR spectrums of DE, **(B)** FTIR spectrums of m-DE, **(C)** FTIR spectrums of m-DE-APTES.

FTIR analyses were conducted to characterize the structure of DE, m-DE, and m-DE-APTES. FTIR spectrum of the particles are shown in Figure 2. Broadband between 3,300–3,500 cm^−1^ (O-H vibration) and the peak at ∼1,630 cm^−1^ (water bending) are caused by the water molecules that remain physically in the DE structure (Zhao et al., 2008; Chen et al., 2013b). The peaks around 1,010–1,030 cm^−1^ consist of asymmetric stretching in Si-O-Si bonds, while the peaks between 790 and 800 cm^−1^ correspond to Al-O-Si stretching vibrations (Sheng et al., 2009). Bands (∼580 cm^−1^) originating from Fe-O bonds following the introduction of magnetite particles into the structure are noteworthy (Chernyshova, 2003; Araujo et al., 2015). The peak at 2,929 cm^−1^ (C-H stretching) results from the APTES molecules.

**FIGURE 3:**
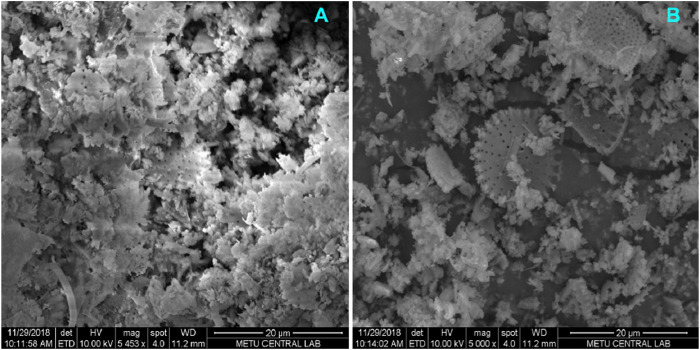
SEM image of **(A)** DE, **(B)** m-DE-APTES.

Scanning electron microscopy was conducted to analyze the structures’ surface morphology (Erol et al., 2019b). The porous structure of DE can be clearly observed in Figure 3. Magnetite particles included in the structure have partially filled the pores. No significant changes were observed in the surface morphology of the particles after APTES modification.

The magnitude of the magnetic properties of m-DE-APTES was determined in this study by ESR and EDX analysis.

**FIGURE 4:**
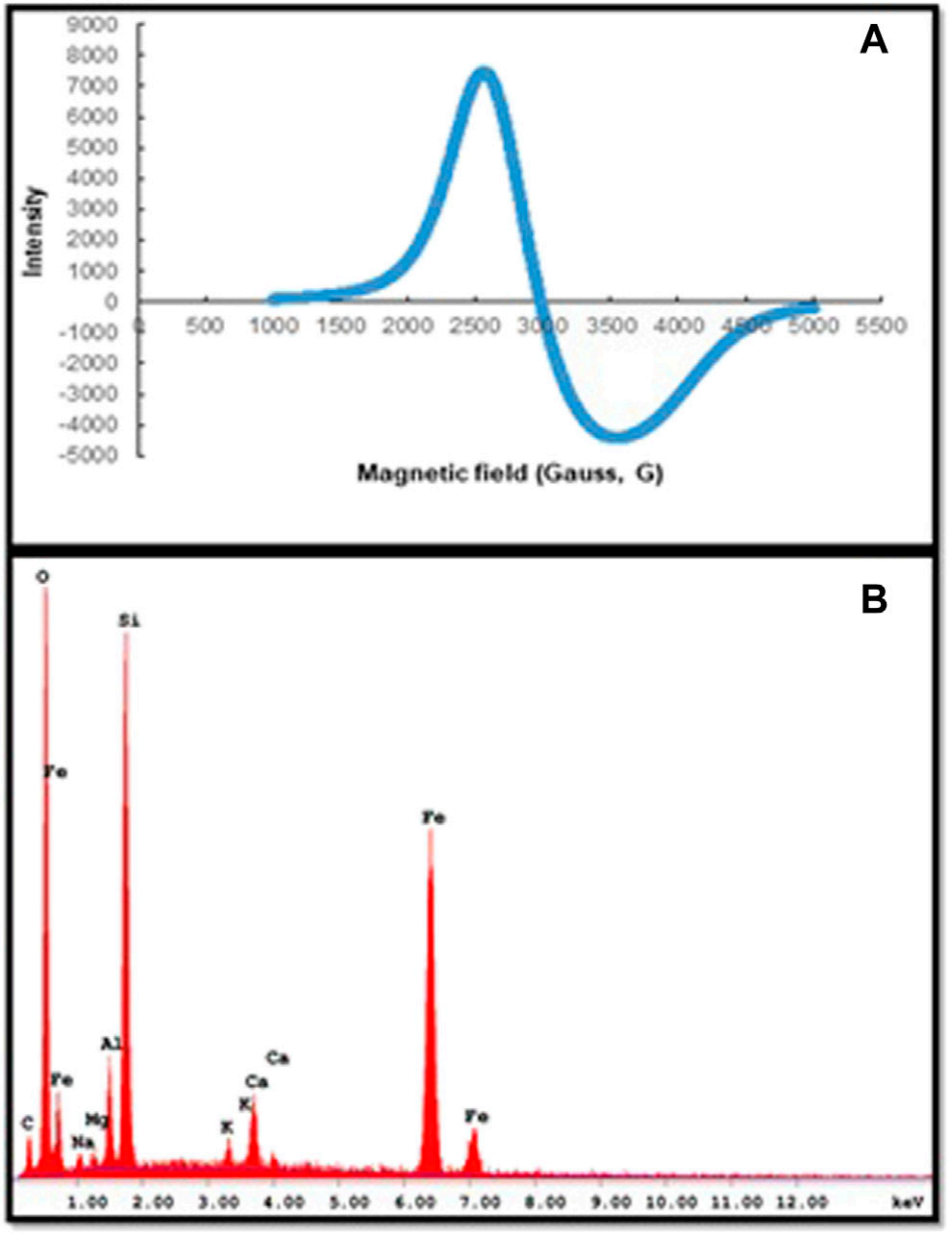
**(A)** ESR spectra of m-DE-APTES, **(B)** EDX spectra of m-DE-APTES.

By the way, g is approximately 2.0, indicating that the magnetic force derives exclusively from uncoupled spins. The g factor of m-DE-APTES was found by calculating 2.32 using Figure 4A. This value indicates that the structure has a local magnetic field due to the precipitation of magnetite particles into the pores. Simultaneously, we can attest that atoms have an orbital effect on the magnetic moment. Additionally, EDX analyses revealed that magnetite particles entered the DE structure and that the APTES molecule was generally bound to the surface. When the EDX spectra of the particles are examined, it is possible to see the Fe atoms from the magnetite and the carbon atoms from the APTES (Figure 4B) (Erol et al., 2019b).

**FIGURE 5:**
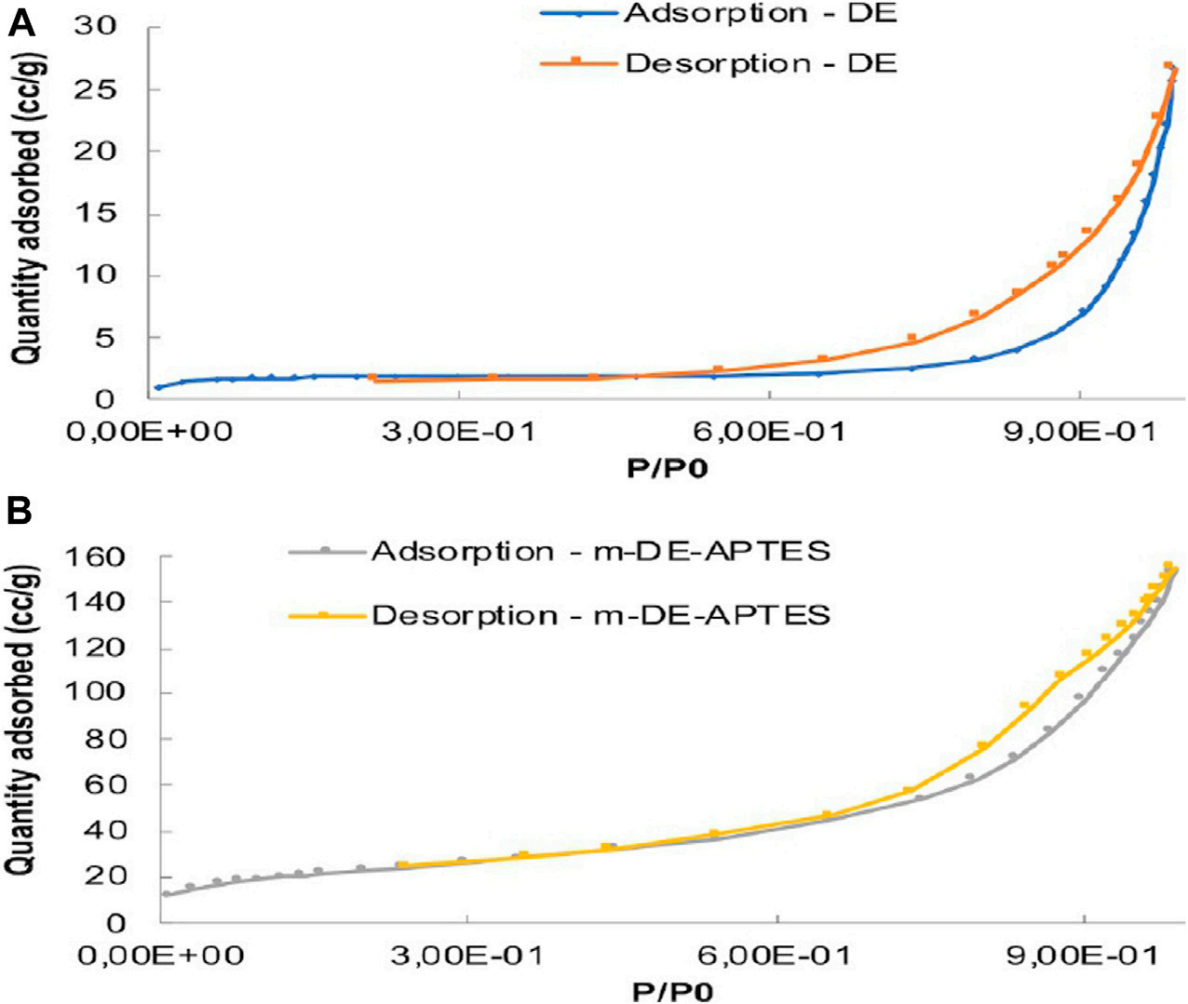
N_2_ adsorption-desorption isotherms of **(A)** DE **(B)** m-DE-APTES.

BET analyses of m-DE-APTES and DE were performed. According to BET analyses, surface area and total pore volume of DE increased proportionately with the addition of magnetite and APTES molecules. In contrast, the average pore diameter decreased (BET analyses of DE and m-DE-APTES are depicted in Supplementary Table S1). The results indicate that DE has been successfully modified with the magnetite crystals precipitating into large pores of diatomite structure which decreased the average pore diameter. According to the IUPAC classification, DE follows the type II b companion model, as shown in Figure 5 (Alacabey et al., 2020).

### Adsorption-Desorption Studies

**FIGURE 6:**
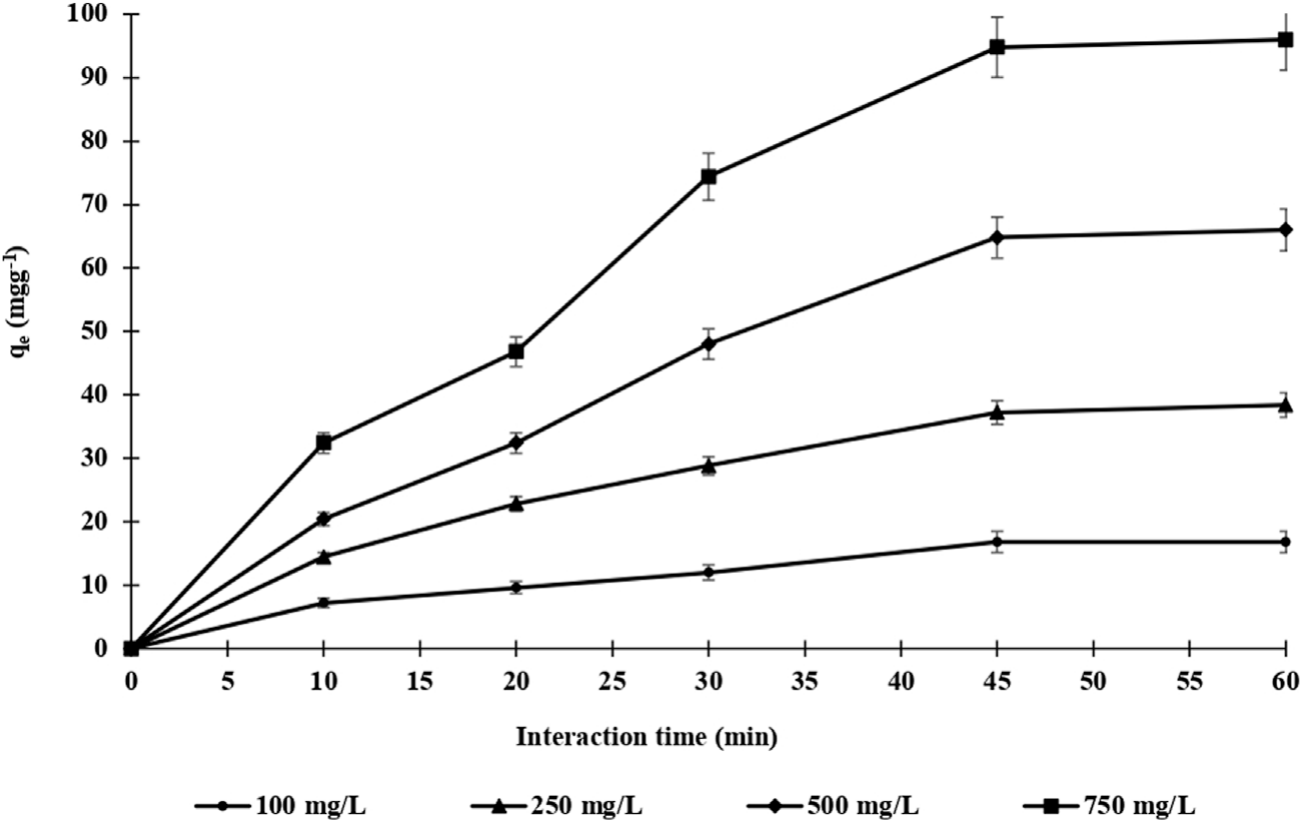
The effect of initial endosulfan concentration on the adsorption capacity of m-DE-APTES.

**FIGURE 7:**
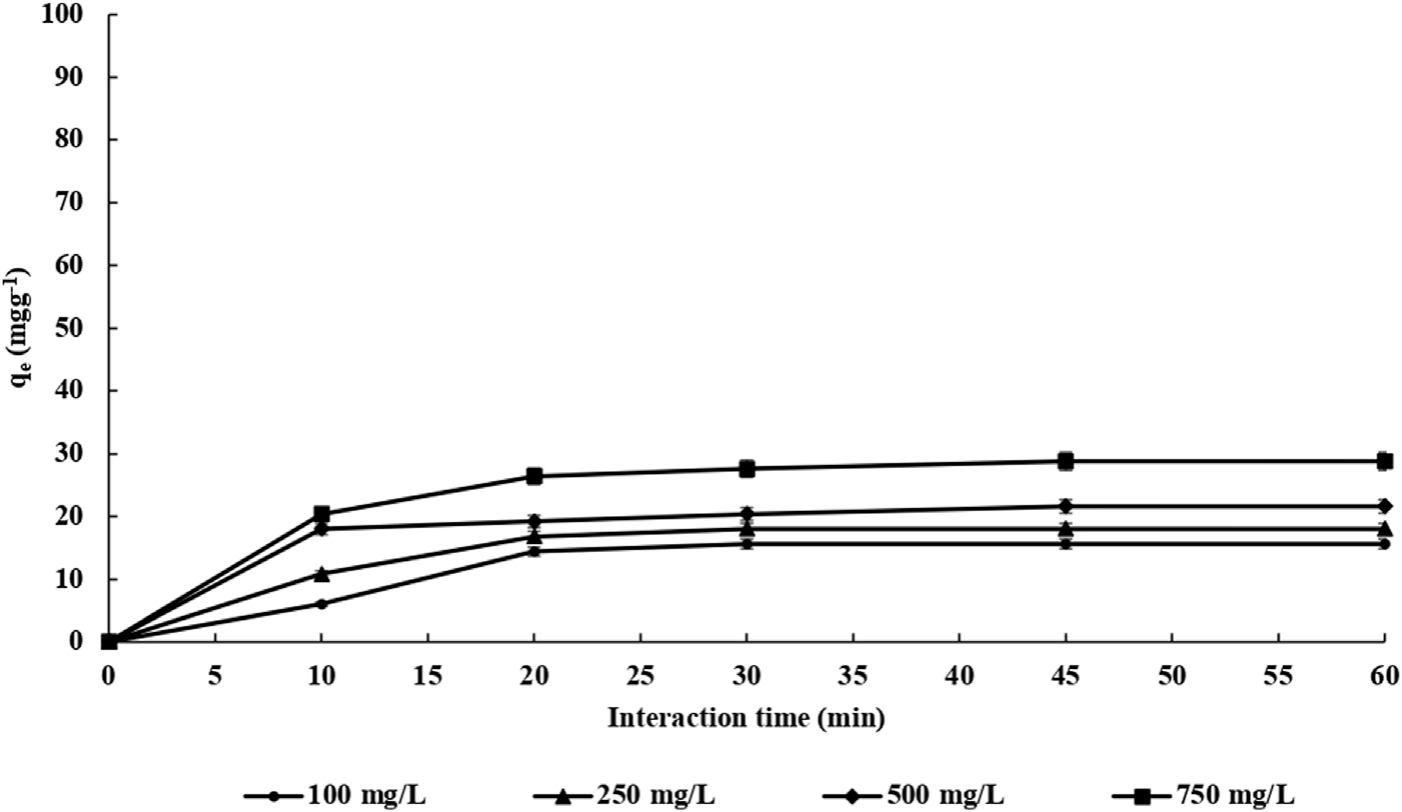
The effect of interaction time on the adsorption capacity of DE-APTES.

The adsorption time of endosulfan adsorption on both m-DE-APTES and DE-APTES was investigated via the batch system (Erol et al., 2020; Erol and Uzun, 2017; Erol, 2017a; Erol, 2017b; Kireç et al., 2021; Erol et al., 2021; Erol, 2017c; Tosun Satir and Erol, 2021) at 25°C. Throughout the experiments, four different endosulfan concentrations (100.0–750.0 mg L^−1^) were used at various times (10–60 min) (Figure 6). As shown in Figure 5, the adsorption capacity of m-DE-APTES for endosulfan increased within 45 min and then reached equilibrium at the 60th minute. Simultaneously, the adsorption capacity was determined to be 97.2 and 16.6 mg g^−1^ with an initial endosulfan concentration of 750 and 100 mg L^−1^, respectively. On the other hand, the adsorption capacity of non-magnetic diatomites (DE-APTES) was found to be 28.8 and 15.6 mg g^−1^ with an initial endosulfan concentration of 750 and 100 mg L^−1^, respectively (Figure 7). The higher adsorption capacity of m-DE-APTES compared with DE-APTES may be due to the greater effective surface area and pore size (Toprak and Kopac, 2019; Zhao et al., 2019).

**FIGURE 8:**
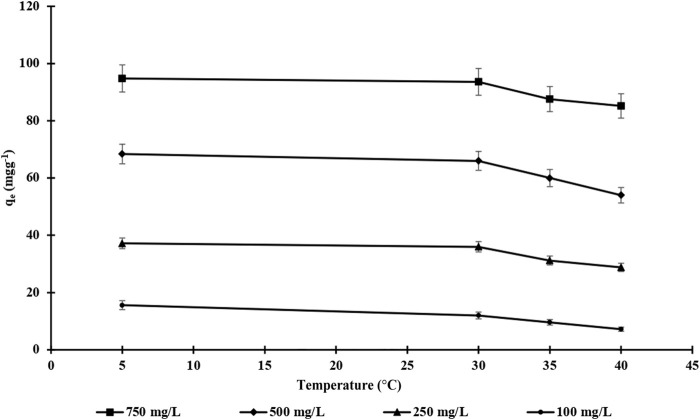
The effect of endosulfan concentration and temperature on adsorption Capacity of m-DE-APTES.

The effect of temperature was also investigated by altering the temperature from 4 to 40°C while using different endosulfan concentrations (100–750 mg L^−1^) (Figure 8). It is clearly shown Figure 8 that the adsorption capacity of m-DE-APTES decreased with increasing temperature. This phenomenon indicates that when the temperature rises, the strength of the bond that provides adsorption interaction weakens. It can also be concluded that, consistent with these results, the dominant interactions between the APTES groups bound to the DE and endosulfan have an electrostatic nature (Erol and Uzun, 2017).

**FIGURE 9:**
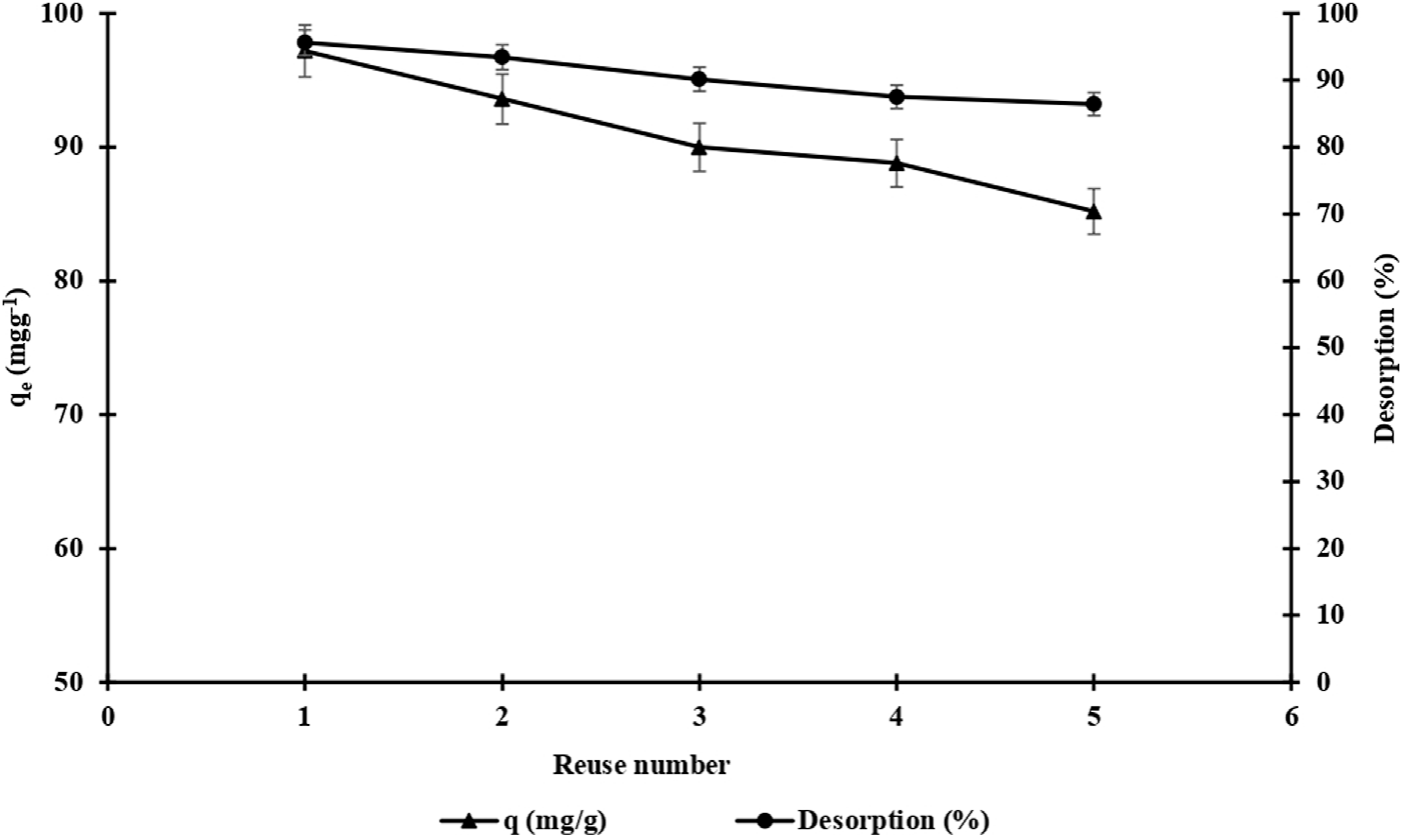
Reusability of m-DE-APTES.

The adsorption-desorption-regeneration cycle repeated five times was performed with identical m-DE-APTES to investigate the reusability of the m-DE-APTES,. The NaCl (1.0 mol L^−1^, pH: 7.0) solution was used as the desorption agent, and samples were mixed in this solution at a mixing rotation rate of 125 rpm at room temperature for 1 h. The calculations revealed that the adsorption capacity was 97.2 mg g^−1^ in the 1st cycle and 85.2 mg g^−1^ in the 5th cycle (Figure 9). At the end of five cycles, the 12.4% decrease in adsorption capacity is a decent value. It is worth noting that the reusability performance of m-DE-APTES is relatively high. Moreover, desorption performance of 90.7% was achieved at the end of five cycles. These findings suggest that the adsorption interaction is reversible (Erol and Uzun, 2017).

Linear [Freundlich, Langmuir, Temkin, and Dubinin–Radushkevich (D-R)] and non-linear (Redlich-Peterson, Sips, and Toth) adsorption isotherm models were used to explain the structure (surface properties, adsorption mechanism and capacity) of endosulfan adsorption on m-DE-APTES. The solver plugin in Microsoft Excel and Microsoft’s spreadsheet was utilized to solve the non-linear equations of the applied isotherms. The equations and parameters of the linear isotherm models and non-linear isotherm models that were used are shown in Table 1 with the relevant references.

**TABLE 1:** Linear adsorption isotherms and Non-Linear adsorption isotherms.

Isotherm	Linear Form	Constants	References
Freundlich	$\ln q_{e}=\;\ln K_{F}+\frac{1}{n}\ln C_{e\;}$	KF [(mgg^−1^) (Lmg^−1^)]^1/n^: adsoption capacity n: heterogeneity factor	Alacabey et al. (2021)
Langmuir	$\frac{1}{q_{e}}=\;\left(\frac{1}{K_{L}q_{\max}}\right)\frac{1}{C_{e}}+\;\frac{1}{q_{\max}}$	q_max_ (mg g^−1^): max adsoption capacity, K_L_ (L mg^−1^): adsorption equilibrium constant	Alacabey, (2022a)
Temkin	q_e_ = B_T_lnK_T_ + B_T_lnC_e_, B_T_= (RT)/b_T_	B_T_ (J mol^−1^): the variation of adsorption energy, b_T_: Temkin isotherm constant, K_T_ (L mg^−1^): Equilibrium binding constant	Alacabey, (2022b)
Dubinin–Radushkevich	lnq_e_ = lnq_m_—K_ad_ε^2^, $\varepsilon =RT\ln\left[1+\;\frac{1}{C_{e}}\right]$ $E=\;\left[\frac{1}{\sqrt{2K_{ad}}}\right]$	q_m_ (mg g^−1^): D–R adsorption capacity, K_ad_ (mol^2^ kJ^−2^): constant due to adsorption energy, ε (kJ mol^−1^): D–R isotherm constant (polanyi potential), E (kJ mol^−1^): D–R adsorption energy, R (8.314 J K^−1^ mol^−1^): universal gas constant and T(K): absolute temperature	Riza et al. (2011)
**Isotherm**	**Non-Linear form**	**Constants**	**References**
Redlich-Peterson	$q_{e}=\;\frac{K_{R}C_{e}}{1+a_{R}C_{e}^{b_{R}}}$	K_R_ (L g^−1^):_:_ Redlich-Peterson isotherm constant, a_R_ (L g^−1^): Redlich-Peterson isotherm constant b_R_: Redlich-Peterson isotherm constant	Yadav and Singh, (2017)
Sips	$q_{e}=\;\frac{\;q_{m}a_{s}C_{e}^{1/n}}{1+\;a_{S}C_{e}^{1/n}}$	q_m_ (mg g^−1^): Maximum adsorption capacity, a_S_ (L g^−1^): Term related to adsorption energy, n: Sips isotherm exponent	Mahmoud et al. (2020)
Toth	$q_{e}=\;\frac{q_{m}C_{e}}{{\left(K_{t}+\;C_{e}\right)}^{1/n_{T}}}$	q_m_ (mg g^−1^): max adsorption capacity, K_t_ (mg g^−1^): Toth isotherm constant. n_T_ (L mg^−1^): Toth isotherm exponent	Mahmoud et al. (2020)

Equilibrium data were applied to the equations of linearized Langmuir isotherm models and linearized Freundlich isotherm model. The correlation between the Langmuir isotherm model and the equilibrium data was strong (*R* ^2^), indicating the non-linear adsorption isotherm (Table 2).

**TABLE 2:** Langmuir, Freundlich, Dubinin-Radushkevich (D–R) and Temkin Redlich-Peterson, Sips and Toth adsorption isotherm values and correlation coefficients.

T (K)	Langmuir			Freundlich			
	K_L_ (L mg^−1^)	q_max_ (mg g^−1^)	*R* ^2^	n	1/n	K_F_ [(mgg^−1^) (Lmg^−1^)]^1/n^	*R* ^2^
278	0.0074	114.8789	0.9683	1.4142	0.7071	1.7648	0.9389
303	0.0023	207.1927	0.9904	1.1549	0.8659	0.6991	0.9684
308	0.0006	549.9127	0.9905	1.0222	0.9783	0.3158	0.9410
313	0.0002	1,052.1084	0.9856	1.0059	0.9941	0.2611	0.9157

**Dubinin-Radushkevich (D—R)**				**Temkin**			
**T (K)**	**q_m_ (mol g^−1^)**	**E (kj mol^−1^)**	***R* ^2^**	**B_T_ (j mol^−1^)**	**b_T_ (kj mol^−1^)**	**K_T_ (L mg^−1^)**	***R* ^2^**
278	70.6313	0.0549	0.7276	34.0506	0.0147	0.0540	0.7559
303	70.5315	0.0406	0.7752	39.2325	0.0156	0.0348	0.8087
308	66.8012	0.0309	0.7880	40.2177	0.0157	0.0260	0.7463
313	64.3324	0.0258	0.8010	41.0983	0.0158	0.0221	0.7070

According to Langmuir isotherm theory, the adsorbate material assumes a single layer on a homogeneous adsorbent surface and that all sorption fields are identical and energy equivalent (Vilela et al., 2020). In Table 2, Langmuir isotherm models were determined to be compatible with the Langmuir isotherm in terms of correlation factors and values between 0.9683 ≤ *R* ^2^ ≤ 0.9905. As a result, it shows that the adsorption occurs in monolayer coated and is homogeneous (Alacabey et al., 2020).

R_L_ values indicate that adsorption is unfavorable (R_L_ > 1), linear (R_L_ = 1), appropriate (0 < R_L_ <1) and irreversible (R_L_ = 0) (Sogut and Caliskan, 2017) As shown in Supplementary Table S2, R_L_ values are appropriate because they fall within the range of 0–1.

$$R_{L}=\frac{1}{1+\;\;K_{L}C_{O\;}\;\;\;}$$ (4)

Where K_L_ is the Langmuir constant indicating the adsorption nature and different isotherm shapes, C_0_ and C_e_ refer to endosulfan’s initial and equilibrium mass concentration (Sogut and Caliskan, 2017).

The Freundlich isotherm theory indicates that the adsorption sites on an adsorbent surface are heterogeneous; they consist of different adsorption fields. Generally, n values between 1 and 10 are indicative of good adsorption. The 1/n value is a heterogeneity factor that encompasses values in the range 0–1 (Table 2). The more heterogeneous the surface, the nearer to zero the value of n. The constant n determines the type of process: if n = 1, the adsorption is linear; If *n* < 1, adsorption is a chemical process; If n > 1, the adsorption takes place in a physical process (Alacabey, 2022a).

The Temkin isotherm model (Table 2) was used to determine the heat of adsorption and evaluate the interaction between adsorbent and adsorbate. This isotherm implies that the adsorption heat (the function of temperature) of all molecules of the adsorbent layers will decrease linearly as the process progresses (Dada et al., 2012). Similarly to the Freundlich equation, the Temkin model considers the surface’s heterogeneity (Ghogomu et al., 2013).

The Dubinin-Radushkevich isotherm theory states that adsorption energy E (kJ mol^−1^) provides data about adsorption’s physical and chemical properties. If the E value is between 8–16 kJmol^-1^, it means that sorption occurs primarily via the ion exchange mechanism. Adsorption for E < 8 kJ mol^−1^ values can be explained as having a physical structure. However, if E is greater than 16 kJ mol^−1^, the adsorption mechanism can be explained by chemical interactions (Caliskan et al., 2011). Because the E and B_T_ values in both D-R and Temkin adsorption isotherms are less than 8 kJ mol^−1^, the adsorption mechanism can be explained by physical interaction (Table 2).

The Redlich-Peterson isotherm is a mixed isotherm that combines the properties of the Langmuir and Freundlich isotherms and has three parameters in the experimental equation. *β* value is between 0 and 1. There are two distinct determinant behaviours: Langmuir form for *β* = 1 and Henry’s law form for *β* = 0 (Vijayaraghavan et al., 2006). As shown in Table 3, *β* = 1 for endosulfan adsorption on m-DE-APTES. This indicates that adsorption is more compatible with the Langmuir isotherm.

**TABLE 3:** Redlich-Peterson, Sips and Toth adsorption isotherm values and correlation coefficients.

T (K)	REDLICH-PETERSON				SIPS				TOTH			
	K_R_ (L g^−1^)	a_R_ (L g^−1^)	*β*	*R* ^2^	q_m_ (mg g^−1^)	a_s_ (L g^−1^)	n	*R* ^2^	q_m_ (mg g^−1^)	K_t_ (mg g^−1^)	n_T_	*R* ^2^
278	2.80	0.0280	1	0.8491	100	0.02803	1	0.8491	100	36	1	0.849
303	0.44	0.0015	1	0.8954	1,020	0.00037	1	0.9157	294	670	1	0.895
308	0.30	0.0006	1	0.8210	1,170	0.00024	1	0.8279	466	1,552	1	0.821
313	0.26	0.0005	1	0.7655	1,170	0.00021	1	0.7699	477	1863	1	0.765

The Sips isotherm is a hybrid of the Langmuir and Freundlich isotherms and is more suitable for explaining adsorption on heterogeneous surfaces. Sips isotherm equation is defined by the dimensionless heterogeneity factor a_S_. If a_S_ = 1, the Sips equation is reduced to the Langmuir equation, indicating a homogeneous adsorption process (Sogut and Caliskan, 2017). As shown in Table 3, endosulfan adsorption on m-DE-APTES has an a_S_ value of 1. This result demonstrates that adsorption is a better fit for the Langmuir isotherm.

The Toth adsorption isotherm is a valid model for heterogeneous adsorption derived from potential theory. The values of the Toth isotherm parameters are shown in Table 3. If the n_T_ value is close to 1, the model is reduced to the Langmuir adsorption isotherm equation (Sogut and Caliskan, 2017). This value was determined as one in our study. As a result, Langmuir is more compatible with isotherm.

Adsorption isotherm for endosulfan adsorption onto m-DE-APTES is depicted in Supplementary Figure S1. The m-DE-APTES can be classified as an L-type of Giles based on their initial slopes (Giles et al., 1974a; Giles et al., 1974b).

### Thermodynamic Parameters

The thermodynamic behaviour of endosulfan adsorption on m-DE-APTES was evaluated using the following equations (Alacabey, 2022b). To find the Gibbs free energy of the adsorption process at a specific temperature, the equilibrium constant Kc was calculated with the help of Eq. 5.

$$K_{C}=\frac{C_{a}}{C_{e}}$$ (5)

K_c_: Equilibrium constant.

C_a_: Concentration of substance held by adsorbent (mg L^−1^).

C_e_: Concentration of substance remaining in solution (mg L^−1^)

$$\Delta G^{0}=-R\;T\;InK_{C}$$ (6)

$$\ln K_{c}\;=\;\frac{\Delta S^{o}}{R}-\frac{\Delta H^{o}}{RT}$$ (7)

Here, R (8.314 J mol^−1^ K^−1^) is the ideal gas constant, and T (K) is the temperature in Kelvin. ΔH^o^ is the enthalpy change, and ΔS^o^ is the entropy change in a particular process. If K_c_ is calculated in Eq. 5, the Gibbs free energy of the adsorption is determined in Eq. 6. Eq. 7 can also be used to calculate ΔH^o^ and ΔS^o^.

As a result, ΔH^o^, ΔG^o^, and ΔS^o^ values determined for m-DE-APTES are given in Table 4. The degree of the spontaneity of the adsorption process is determined by the Gibbs free energy (Depci et al., 2011). Positive ΔH^o^ values indicate that adsorption is endothermic, while negative ∆G^o^ values indicate spontaneous adsorption. Additionally, a positive value suggests an increase in randomness at the solid-liquid interface during endosulfan adsorption onto the adsorbent (Caliskan et al., 2011; Vilela et al., 2020). Increased temperature decreases free active adsorption sites, which results in a decrease ΔG^o^ values (Alacabey, 2022b).

**TABLE 4:** Thermodynamic parameters.

C_0_ (mg L^−1^)	ΔH° (kj mol^−1^)	ΔS° (j mol^−1^)	ΔG° (kj mol^−1^)			
			278 K	303 K	308 K	313 K
100	21.377	67.188	−2.576	−1.593	−0.545	−0.031
250	10.870	32.440	−1.755	−1.463	−0.839	−0.430
500	7.302	20.852	−1.437	−1.240	−0.938	−0.529
750	5.528	14.689	−1.401	−1.238	−1.043	−0.773

## Conclusion

In this study, m-DE-APTES were synthesized, and the endosulfan adsorption performance of these particles was investigated. The highest adsorption capacity value was obtained in the conditions of 750 mg L^−1^ endosulfan concentration and 45 min interaction time. The incorporation of the magnetite molecules into the DE increased the structure’s surface area (from 5.62 to 84.22 m^2^ g^−1^), resulting in an increased adsorption capacity for endosulfan. The structure was functionalized with APTES molecules, which enhanced the adsorption capacity and increased the interaction between magnetic DE and endosulfan molecules. In particular, it is believed that there is an electrostatic interaction between the charged groups of the APTES molecule and the oppositely charged groups on the endosulfan. This possible interaction forms the basis of the adsorption mechanism between the adsorbent and pesticide. While an adsorption capacity of 97.2 mg endosulfan g^−1^ was obtained with the m-DE-APTES material, a rate of 90.7% was reached for desorption. A large scale study was performed with linear and non-linear adsorption isotherm models, and as a result, it was determined that the best fit model was the Langmuir adsorption model. Therefore, it can be concluded that the adsorption of endosulfan to m-DE-APTES material occurs in a single layer and that the adsorption mechanism occurs as physical adsorption. As a result, m-DE-APTES are promising materials for removing endosulfan from wastewaters.

## Data Availability

The original contributions presented in the study are included in the article/Supplementary Material, further inquiries can be directed to the corresponding author.

## References

[B1] Acet Ö. Baran T. Erdönmez D. Aksoy N. H. Alacabey İ. Menteş A. (2018). O-carboxymethyl Chitosan Schiff Base Complexes as Affinity Ligands for Immobilized Metal-Ion Affinity Chromatography of Lysozyme. J. Chromatogr. A 1550, 21–27. 10.1016/j.chroma.2018.03.022 29609862

[B2] Al-Shaalan N. H. Ali I. ALOthman Z. A. Al-Wahaibi L. H. Alabdulmonem H. (2019). High Performance Removal and Simulation Studies of Diuron Pesticide in Water on MWCNTs. J. Mol. Liq. 289, 111039. 10.1016/j.molliq.2019.111039

[B3] Alacabey İ. Acet Ö. Önal B. Dikici E. Karakoç V. Gürbüz F. (2021). Pumice Particle Interface: a Case Study for Immunoglobulin G Purification. Polym. Bull. 78, 5593–5607. 10.1007/s00289-020-03392-0

[B4] Alacabey İ. (2022). Adsorptive Removal of Cationic Dye from Aqueous Solutions Using Bardakçı Clay. Int. J. Agric. Environ. Food Sci. 6, 80–90. 10.31015/jaefs.2022.1.12

[B5] Alacabey İ. (2022). Antibiotic Removal from the Aquatic Environment with Activated Carbon Produced from Pumpkin Seeds. Molecules 27, 1380. 10.3390/molecules27041380 35209169 PMC8877137

[B6] Alacabey İ. Kul A. R. Ece Ş. Alkan H. (2020). Van Gölü Doğal Sediment Ve Modifiye Sediment Üzerine Krom (III) Adsorpsiyonu (Izoterm Ve Termodinamik Analiz Çalışması). Dicle Üniversitesi Mühendislik Fakültesi Mühendislik Derg. 11, 1225–1232. 10.24012/dumf.731216

[B7] Ali A. Jain C. K. (1998). Groundwater Contamination and Health Hazards by Some of the Most Commonly Used Pesticides. Curr. Sci. 75, 1011–1014.

[B8] Araujo R. T. Ferreira G. R. Segura T. Souza F. G. Jr Machado F. (2015). An Experimental Study on the Synthesis of Poly(vinyl Pivalate)-Based Magnetic Nanocomposites through Suspension Polymerization Process. Eur. Polym. J. 68, 441–459. 10.1016/j.eurpolymj.2015.05.015

[B9] Armutcu C. Özgür E. Karasu T. Bayram E. Uzun L. Çorman M. E. (2019). Rapid Analysis of Polycyclic Aromatic Hydrocarbons in Water Samples Using an Automated On-Line Two-Dimensional Liquid Chromatography. Water Air Soil Pollut. 230, 1–11. 10.1007/s11270-019-4306-7

[B10] Arnnok P. Burakham R. (2014). Retention of Carbamate Pesticides by Different Surfactant-Modified Sorbents: a Comparative Study. J. Braz. Chem. Soc. 25, 1720–1729. 10.5935/0103-5053.20140167

[B11] Basheer A. A. Ali I. (2018). Stereoselective Uptake and Degradation of (±)-o ,p -DDD Pesticide Stereomers in Water-Sediment System. Chirality 30, 1088–1095. 10.1002/chir.22989 29978905

[B12] Caliskan N. Kul A. R. Alkan S. Sogut E. G. Alacabey İ. (2011). Adsorption of Zinc(II) on Diatomite and Manganese-Oxide-Modified Diatomite: A Kinetic and Equilibrium Study. J. Hazard. Mater. 193, 27–36. 10.1016/j.jhazmat.2011.06.058 21764214

[B13] Chawla P. Kaushik R. Shiva Swaraj V. J. Kumar N. (2018). Organophosphorus Pesticides Residues in Food and Their Colorimetric Detection. Environ. Nanotechnol. Monit. Manag. 10, 292–307. 10.1016/j.enmm.2018.07.013

[B14] Chen J. Y. Lin Y. J. Kuo W. C. (2013). Pesticide Residue Removal from Vegetables by Ozonation. J. Food Eng. 114, 404–411. 10.1016/j.jfoodeng.2012.08.033

[B15] Chen L. Xu J. Hu J. (2013). Removal of U(VI) from Aqueous Solutions by Using Attapulgite/iron Oxide Magnetic Nanocomposites. J. Radioanal. Nucl. Chem. 297, 97–105. 10.1007/s10967-012-2360-3

[B16] Chernyshova I. V. (2003). An *In Situ* FTIR Study of Galena and Pyrite Oxidation in Aqueous Solution. J. Electroanal. Chem. 558, 83–98. 10.1016/S0022-0728(03)00382-6

[B17] Çorman M. E. Armutcu C. Uzun L. Denizli A. (2017). Rapid, Efficient and Selective Preconcentration of Benzo[a]pyrene (BaP) by Molecularly Imprinted Composite Cartridge and HPLC. Mater. Sci. Eng. C 70, 41–53. 10.1016/j.msec.2016.08.040 27770911

[B18] Dada A. Olalekan A. Olatunya A. Dada O. (2012). Langmuir, Freundlich, Temkin and Dubinin–Radushkevich Isotherms Studies of Equilibrium Sorption of Zn2+ unto Phosphoric Acid Modified Rice Husk. IOSR J. Appl. Chem. 3, 38–45.

[B19] Depci T. Alkan S. Kul A. Önal Y. Alacabey I. Dişli E. (2011). Characteristic Properties of Adsorbed Catalase onto Activated Carbon Based Adiyaman Lignite. Fresenius Environ. Bull. 20 (9), 2371–2378.

[B20] Donia A. M. Atia A. A. Hussien R. A. Rashad R. T. (2012). Comparative Study on the Adsorption of Malathion Pesticide by Different Adsorbents from Aqueous Solution. Desalination Water Treat. 47, 300–309. 10.1080/19443994.2012.696419

[B21] Edathil A. A. Shittu I. Hisham Zain J. Banat F. Haija M. A. (2018). Novel Magnetic Coffee Waste Nanocomposite as Effective Bioadsorbent for Pb(II) Removal from Aqueous Solutions. J. Environ. Chem. Eng. 6, 2390–2400. 10.1016/j.jece.2018.03.041

[B22] Erol B. Erol K. Gökmeşe E. (2019). The Effect of the Chelator Characteristics on Insulin Adsorption in Immobilized Metal Affinity Chromatography. Process Biochem. 83, 104–113. 10.1016/j.procbio.2019.05.009

[B23] Erol K. Bolat M. Tatar D. Nigiz C. Köse D. A. (2020). Synthesis, Characterization and Antibacterial Application of Silver Nanoparticle Embedded Composite Cryogels. J. Mol. Struct. 1200, 127060. 10.1016/j.molstruc.2019.127060

[B24] Erol K. (2016). DNA Adsorption via Co(II) Immobilized Cryogels. J. Macromol. Sci. Part A 53, 629–635. 10.1080/10601325.2016.1212310

[B25] Erol K. Köse K. Köse D. A. Sızır Ü. Tosun Satır İ. Uzun L. (2016). Adsorption of Victoria Blue R (VBR) Dye on Magnetic Microparticles Containing Fe(II)-Co(II) Double Salt. Desalination Water Treat. 57, 9307–9317. 10.1080/19443994.2015.1030708

[B26] Erol K. (2017). Polychelated Cryogels: Hemoglobin Adsorption from Human Blood. Artif. Cells, Nanomedicine, Biotechnol. 45, 31–38. 10.1080/21691401.2016.1215326 27684101

[B27] Erol K. (2017). Synthesis, Characterization and Chromatographic Applications of Antimicrobial Cryogels. Hjbc 2, 187–195. 10.15671/HJBC.2017.151

[B28] Erol K. Tatar D. Veyisoğlu A. Tokatlı A. (2021). Antimicrobial Magnetic Poly(GMA) Microparticles: Synthesis, Characterization and Lysozyme Immobilization. J. Polym. Eng. 41, 144–154. 10.1515/polyeng-2020-0191

[B29] Erol K. (2017). The Adsorption of Calmoduline via Nicotinamide Immobilized Poly(HEMA-GMA) Cryogels. J. Turkish Chem. Soc. Sect. A Chem. 4, 133. 10.18596/jotcsa.287321

[B30] Erol K. Uzun L. (2017). Two-step Polymerization Approach for Synthesis of Macroporous Surface Ion-Imprinted Cryogels. J. Macromol. Sci. Part A 54, 867–875. 10.1080/10601325.2017.1342519

[B31] Erol K. Yıldız E. Alacabey İ. Karabörk M. Uzun L. (2019). Magnetic Diatomite for Pesticide Removal from Aqueous Solution via Hydrophobic Interactions. Environ. Sci. Pollut. Res. 26, 33631–33641. 10.1007/s11356-019-06423-0 31587166

[B32] Ghogomu J. Noufame T. Ketcha M. Ndi N. (2013). Removal of Pb(II) Ions from Aqueous Solutions by Kaolinite and Metakaolinite Materials. Bjast 3, 942–961. 10.9734/BJAST/2013/4384

[B33] Ghuffar S. Irshad G. Naz F. Khan M. A. (2021). Studies of Penicillium Species Associated with Blue Mold Disease of Grapes and Management through Plant Essential Oils as Non-hazardous Botanical Fungicides. Green Process. Synthesis 10, 021–036. 10.1515/gps-2021-0007

[B34] Giles C. H. D'Silva A. P. Easton I. A. (1974). A General Treatment and Classification of the Solute Adsorption Isotherm Part. II. Experimental Interpretation. J. colloid interface Sci. 47, 766–778. 10.1016/0021-9797(74)90253-7

[B35] Giles C. H. Smith D. Huitson A. (1974). A General Treatment and Classification of the Solute Adsorption Isotherm. I. Theoretical. J. colloid interface Sci. 47, 755–765. 10.1016/0021-9797(74)90252-5

[B36] Gimsing A. Sorensen J. Strobel B. Hansen H. (2007). Adsorption of Glucosinolates to Metal Oxides, Clay Minerals and Humic Acid. Appl. Clay Sci. 35, 212–217. 10.1016/j.clay.2006.08.008

[B37] Hernández A. F. Parrón T. Tsatsakis A. M. Requena M. Alarcón R. López-Guarnido O. (2013). Toxic Effects of Pesticide Mixtures at a Molecular Level: Their Relevance to Human Health. Toxicology 307, 136–145. 10.1016/j.tox.2012.06.009 22728724

[B38] Iizuka T. Yahata M. Shimizu A. (2013). Potential Mechanism Involved in Removal of Hydrophobic Pesticides from Vegetables by Hydrostatic Pressure. J. Food Eng. 119, 1–6. 10.1016/j.jfoodeng.2013.05.006

[B39] Johnson T. Brineman R. Schultze C. Barkovskii A. L. (2020). Efficient Removal of Bacteria from Aqueous Media with Kaolinite and Diatomaceous Earth Products. J. Appl. Microbiol. 129, 466–473. 10.1111/jam.14642 32180297

[B40] Kabiri S. Tran D. N. H. Azari S. Losic D. (2015). Graphene-diatom Silica Aerogels for Efficient Removal of Mercury Ions from Water. ACS Appl. Mat. Interfaces 7, 11815–11823. 10.1021/acsami.5b01159 25835089

[B41] Karanasios E. Tsiropoulos N. G. Karpouzas D. G. (2012). On-farm Biopurification Systems for the Depuration of Pesticide Wastewaters: Recent Biotechnological Advances and Future Perspectives. Biodegradation 23, 787–802. 10.1007/s10532-012-9571-8 23054187

[B42] Kireç O. Alacabey İ. Erol K. Alkan H. (2021). Removal of 17β-Estradiol from Aqueous Systems with Hydrophobic Microspheres. J. Polym. Eng. 41, 226–234. 10.1515/polyeng-2020-0150

[B43] Köse K. Erol K. Emniyet A. A. Köse D. A. Avcı G. A. Uzun L. (2015). Fe(II)-Co(II) Double Salt Incorporated Magnetic Hydrophobic Microparticles for Invertase Adsorption. Appl. Biochem. Biotechnol. 177, 1025–1039. 10.1007/s12010-015-1794-9 26265396

[B44] Kucuk F. Sismanoglu S. Kanbur Y. Tayfun U. (2020). Effect of Silane-Modification of Diatomite on its Composites with Thermoplastic Polyurethane. Mater. Chem. Phys. 256, 123683. 10.1016/j.matchemphys.2020.123683

[B45] Kul A. Alacabey İ. Kılıç N. Ç. (2010). Removal of Cobalt Ions from Aqueous Solution by Diatomite. Hacettepe J. Biol. Chem. 38, 85–93.

[B46] Kupski L. Salcedo G. M. Caldas S. S. de Souza T. D. Furlong E. B. Primel E. G. (2019). Optimization of a Laccase-Mediator System with Natural Redox-Mediating Compounds for Pesticide Removal. Environ. Sci. Pollut. Res. 26, 5131–5139. 10.1007/s11356-018-4010-y 30607853

[B47] Mahmoud M. E. El-Said G. F. Rashedy I. R. K. Abdelfattah A. M. (2020). Assembly and Implementation of an Eco-Friendly Marine Nanosediment for Adsorptive Removal of Heptavalent Manganese: Adsorption Isotherm, Thermodynamic and Kinetics Studies. Powder Technol. 359, 247–260. 10.1016/j.powtec.2019.09.063

[B48] Marican A. Durán-Lara E. F. (2018). A Review on Pesticide Removal through Different Processes. Environ. Sci. Pollut. Res. 25, 2051–2064. 10.1007/s11356-017-0796-2 29185220

[B49] Massart R. (1981). Preparation of Aqueous Magnetic Liquids in Alkaline and Acidic Media. IEEE Trans. Magn. 17, 1247–1248. 10.1109/tmag.1981.1061188

[B50] Matsushita T. Morimoto A. Kuriyama T. Matsumoto E. Matsui Y. Shirasaki N. (2018). Removals of Pesticides and Pesticide Transformation Products during Drinking Water Treatment Processes and Their Impact on Mutagen Formation Potential after Chlorination. Water Res. 138, 67–76. 10.1016/j.watres.2018.01.028 29573630

[B51] Michen B. Meder F. Rust A. Fritsch J. Aneziris C. Graule T. (2012). Virus Removal in Ceramic Depth Filters Based on Diatomaceous Earth. Environ. Sci. Technol. 46, 1170–1177. 10.1021/es2030565 22191487

[B52] Mohan V. B. Jayaraman K. Bhattacharyya D. (2020). Brunauer-Emmett-Teller (BET) Specific Surface Area Analysis of Different Graphene Materials: A Comparison to Their Structural Regularity and Electrical Properties. Solid State Commun. 320, 114004. 10.1016/j.ssc.2020.114004

[B53] Mu Y. Cui M. Zhang S. Zhao J. Meng C. Sun Q. (2018). Comparison Study between a Series of New Type Functional Diatomite on Methane Adsorption Performance. Microporous Mesoporous Mater. 267, 203–211. 10.1016/j.micromeso.2018.03.037

[B54] Rasolonjatovo M. A. Cemek M. Cengiz M. F. Ortaç D. Konuk H. B. Karaman E. (2017). Reduction of Methomyl and Acetamiprid Residues from Tomatoes after Various Household Washing Solutions. Int. J. food Prop. 20, 2748–2759. 10.1080/10942912.2016.1250099

[B55] Reynoso Varela A. Vázquez Contreras F. P. de los Santos Villalobos S. Alvarez Valencia L. H. Ulloa Mercado R. G. Serrano Palacios D. (2021). Removal of Endosulfan in a Sequencing Batch Reactor: Addition of Granular Activated Carbon as Improvement Strategy. Water Environ. J. 35, 390–401. 10.1111/wej.12637

[B56] Riza K. A. Tolga D. Ihsan A. Salih A. Yunus O. (2011). Equilibrium, Kinetic and Thermodynamic Studies of Nickel Adsorption onto Natural and Modified Kaolinites. Fresenius Environ. Bull. 20, 1155–1166.

[B57] Robertson L. J. Gjerde B. K. Opsahl M. (2003). Removal of Parasitic Protozoa from Water Using a Mobile Water Filtration Apparatus Intended for Field Use by Military or Emergency Personnel. Mil. Med. 168, 53–56. 10.1093/milmed/168.1.5310.1093/miled.168.1.53 12546247

[B58] Sheng G. Wang S. Hu J. Lu Y. Li J. Dong Y. (2009). Adsorption of Pb(II) on Diatomite as Affected via Aqueous Solution Chemistry and Temperature. Colloids Surfaces A Physicochem. Eng. Aspects 339, 159–166. 10.1016/j.colsurfa.2009.02.016

[B59] Sogut E. G. Caliskan N. (2017). Isotherm and Kinetic Studies of Pb (II) Adsorption on Raw and Modified Diatomite by Using Non-linear Regression Method. Fresenius Environ. Bull. 26, 2721–2729.

[B60] Toprak A. Kopac T. (2019). Effect of Surface Area and Micropore Volume of Activated Carbons from Coal by KOH, NaOH and ZnCl2 Treatments on Methane Adsorption. Int. J. Chem. React. Eng. 17. 10.1515/ijcre-2018-0146

[B61] Tosun Satir İ. Erol K. (2021). Calcined Eggshell for Removal of Victoria Blue R Dye from Wastewater Medium by Adsorption. J. Turkish Chem. Soc. Sect. A Chem. 8, 31–40. 10.18596/jotcsa.760083

[B62] Tsai W.-T. Lai C.-W. Hsien K.-J. (2006). Characterization and Adsorption Properties of Diatomaceous Earth Modified by Hydrofluoric Acid Etching. J. Colloid Interface Sci. 297, 749–754. 10.1016/j.jcis.2005.10.058 16364353

[B63] Vijayaraghavan K. Padmesh T. Palanivelu K. Velan M. (2006). Biosorption of Nickel(II) Ions onto Sargassum Wightii: Application of Two-Parameter and Three-Parameter Isotherm Models. J. Hazard. Mater. 133, 304–308. 10.1016/j.jhazmat.2005.10.016 16297540

[B64] Vilela P. B. Dalalibera A. Becegato V. A. Paulino A. T. (2020). Single-Component and Multi-Component Metal Abatement in Water Using a Hydrogel Based on Chitosan: Characterization, Isotherm, Kinetic, and Thermodynamic Results. Water Air Soil Pollut. 231, 1–14. 10.1007/s11270-020-04873-8

[B65] Wu C. D. Zhang J. Y. Wang L. He M. H. (2013). Removal of Aniline and Phenol from Water Using Raw and Aluminum Hydroxide-Modified Diatomite. Water Sci. Technol. 67, 1620–1626. 10.2166/wst.2013.038 23552253

[B66] Xie F. Xie Z. Zhou B. Li L. Zhou X. Fan Q. (2020). Characteristics and Health Risk Assessment of Organochlorine Pesticides (OCPs) Residues along Sino-Russian Boundary River. Water Air Soil Pollut. 231, 1–12. 10.1007/s11270-020-04877-4

[B67] Yadav M. Singh N. K. (2017). Isotherm Investigation for the Sorption of Fluoride onto Bio-F: Comparison of Linear and Non-linear Regression Method. Appl. Water Sci. 7, 4793–4800. 10.1007/s13201-017-0602-9

[B68] Yuan P. Liu D. Fan M. Yang D. Zhu R. Ge F. (2010). Removal of Hexavalent Chromium [Cr(VI)] from Aqueous Solutions by the Diatomite-Supported/unsupported Magnetite Nanoparticles. J. Hazard. Mater. 173, 614–621. 10.1016/j.jhazmat.2009.08.129 19748178

[B69] Zhao D. Feng S. Chen C. Chen S. Xu D. Wang X. (2008). Adsorption of Thorium(IV) on MX-80 Bentonite: Effect of pH, Ionic Strength and Temperature. Appl. Clay Sci. 41, 17–23. 10.1016/j.clay.2007.09.012

[B70] Zhao J. Qin Y. Shen J. Zhou B. Li C. Li G. (2019). Effects of Pore Structures of Different Maceral Compositions on Methane Adsorption and Diffusion in Anthracite. Appl. Sci. 9, 5130. 10.3390/app9235130

[B71] Zheng X. Dou J. Yuan J. Qin W. Hong X. Ding A. (2017). Removal of Cs+ from Water and Soil by Ammonium-Pillared montmorillonite/Fe3O4 Composite. J. Environ. Sci. 56, 12–24. 10.1016/j.jes.2016.08.019 28571846

