# Supplementary material for: Endosulfan Elimination Using Amine-Modified Magnetic Diatomite as an Adsorbent
Source: Front Chem. 2022 May 26;10:907302. doi: 10.3389/fchem.2022.907302 (PMC9205645; doi:10.3389/fchem.2022.907302)
Supplement: Supplementary file 1 [file Table1.DOCX]

**Table 1.** Summary of BET analyses.

| Particles type | Surface Area (m^2^/g) | Total Pore Volume (cm^3^/g) | Average Pore Size (Å) |
| --- | --- | --- | --- |
| DE | 5.62 | 0.02 | 144.35 |
| m-DE-APTES | 84.22 | 0.19 | 90.92 |

**Table 2.** R_L_ values for Langmuir isotherm.

| T (K) | | | | |
| --- | --- | --- | --- | --- |
| C_0_ (mgL^-1^) | 278 | 303 | 308 | 313 |
|  | R_L_ | R_L_ | R_L_ | R_L_ |
| 100 | 0.5741 | 0.8108 | 0.9477 | 0.9759 |
| 250 | 0.3503 | 0.6315 | 0.8787 | 0.9420 |
| 500 | 0.2123 | 0.4615 | 0.7837 | 0.8903 |
| 750 | 0.1523 | 0.3636 | 0.7072 | 0.8440 |

**Figure 1.** Giles adsorption isotherm for endosulfan sorption onto m-DE-APTES.
